# 305 Increasing Nursing Burn Care Knowledge Through a Podcast Platform

**DOI:** 10.1093/jbcr/irad045.280

**Published:** 2023-08-29

**Authors:** Brianna Weintraub, Scott W Mueller, Arek J Wiktor

**Affiliations:** UCHealth University of Colorado Hospital Burn Center, Lakewood, Colorado; University of Colorado Burn and Frostbite Center, Aurora, Colorado; University of Colorado, Aurora, Colorado

## Abstract

**Introduction:**

Various educational methods and platforms have been developed to teach burn care such as Advanced Burn Life Support, Fundamentals of Burn Care, e-platforms, and supplementary lectures. However, there are nuances to each burn centers’ practices that general knowledge courses do not address. This study investigates the usefulness of using a burn unit podcast as a platform to provide educational delivery of clinical explanations aimed at improving nursing staff knowledge and understanding of clinical burn care.

**Methods:**

A seven-episode podcast curriculum was developed at our ABA verified burn center focusing on seven core topic areas: burn evaluation; burn resuscitation; inhalation injury; wound care/pain management; psychology/nutrition; exfoliative/electrical injuries; frostbite. Each podcast ranged from 14-35 minutes led by burn attending moderators. Baseline data was obtained via an 18 question pre-test completed by participants prior to gaining access to the podcast. A post-test was administered after listening to episodes, comprised of different matched questions. Participant feedback survey was also given. A matched pair analysis of pre and post test results was performed utilizing paired t-test to measure significance.

**Results:**

Over the course of two years, 18 nurses (RN) with varying levels of experience participated in the study. Overall post podcast test score averages significantly improved from 0.77 pre to 0.90 post (P< 0.01). Specific knowledge areas of burn evaluation (0.76 pre, 0.93 post), resuscitation (0.38 pre, 0.88 post), and frostbite (0.69 pre to 0.97 post), were all significantly improved post podcast (p< 0.01). Wound care/pain management (0.88 pre, 0.9 post), psychology/nutrition (0.85 pre, 0.88 post) were not significantly improved. Exfoliative/electrical injuries demonstrated non-significant decrease in scores (0.94 pre, 0.83 post). 93% of RNs strongly agreed that the podcast speaker(s) were knowledgeable and engaged. 86.66% of RNs agreed that the interactive teaching strategy enhanced learning. Additional feedback noted that the podcast was, “entertaining,” “a fun way to learn,” and “a great way to introduce new information.”

**Conclusions:**

Unit specific podcast can improve burn knowledge while engaging the adult learner. Analysis of results can help educators focus on areas that need further attention and emphasis. Nursing feedback revealed that a flexible method of electronic content delivery engaged and entertained the learner while increasing the effectiveness of knowledge attained.

**Applicability of Research to Practice:**

Any burn center focused on education for adult learners as an adjunct to traditional means of learning.

